# Is Pathologic Near-Total Regression an Appropriate Indicator of a Good Response to Preoperative Chemoradiotherapy Based on Oncologic Outcome of Disease?

**DOI:** 10.1097/MD.0000000000002257

**Published:** 2015-12-18

**Authors:** Jee Yeon Kim, In Ja Park, Seung Mo Hong, Jong Lyul Lee, Yong Sik Yoon, Chan Wook Kim, Seok-Byung Lim, Jung Bok Lee, Chang Sik Yu, Jin Cheon Kim

**Affiliations:** From the Department of Colon and Rectal Surgery (JYK, IJP, JLL, YSY, CWK, S-BL, CSY, JCK), Department of Pathology (SMH), and Department of Clinical Epidemiology and Biostatistics, University of Ulsan College of Medicine and Asan Medical Center, Seoul, Korea (JBL).

## Abstract

We evaluated the oncologic outcomes of patients with rectal cancer who demonstrated pathologic near-total regression (NTR) after preoperative chemoradiotherapy (PCRT) and compared with total regression (TR).

Pathologic NTR in rectal cancer by tumor regression grade (TRG) is usually considered to indicate a good response, when evaluating tumor response to PCRT.

We retrospectively analyzed the outcomes in 263 patients who received PCRT for advanced T3/4 or N+ rectal cancer followed by radical resection. Patients were diagnosed with TR (n = 132) or NTR (n = 131) according to the TRG. Recurrence-free survival (RFS) was evaluated and compared between groups. For evaluating the consistency between the result and previously published data, meta-analysis for summing up survival curve was performed using generalized linear mixed model.

ypT status was heterogeneous in the NTR group as follows; 3 Tis (2.3%), 21 T1 (16%), 72 T2 (55%), and 35 T3 (26.7%). Metastatic lymph nodes were more frequently found in the NTR group (6.8% in TR vs 24.4% in NTR patients; *P* = 0.003). The cumulative recurrence rate was higher in the NTR group (19.8% vs 6.1%; *P* = 0.003). The 5-year RFS was lower in the NTR group (94% vs 77.8%; *P* = 0.001). Significant differences in the RFS rate were found in comparison with the published literature.

Based on differences in the oncologic outcomes between the TR and NTR groups, it might not be suitable to use NTR as an indicator of good response to PCRT together with TR.

## Abstract 2

Supplemental Digital Content is available in the text

## INTRODUCTION

The paradigm of clinical medicine is evolving with the concept of precision medicine, which is an integrated effort to treat patients based on their individual characteristics and not by a routine practice guideline. Predicting responses to preoperative chemoradiotherapy (PCRT) is a core step in applying precision medicine to rectal cancer treatment.

Current guidelines suggest PCRT followed by surgery as the standard treatment for all locally advanced rectal cancer patients,^1^ but the degree of tumor response to radiation therapy varies and ranges from complete eradication of the primary tumor to minimal or no radiation-related changes. The benefit of PCRT is optimized in patients who have highly responsive tumor to either radiation or chemotherapy and those patients who have certain clinical characteristics that are associated with superior treatment outcome.^2–4^ However, the available data are insufficient regarding patients in which the tumor response is not sustained and salvage resection is subsequently required due to locoregional failure.^4^

Predicting the tumor response to PCRT will lead to different clinical decisions. We will be able to recommend personalized treatment modalities in different sequences according to their predicted response to PCRT. For example, for patients who are unlikely to respond to PCRT and thus do not seem to benefit from neoadjuvant treatment,^5^ surgery could be performed without delay. Therefore, in recent years, there has been great interest in identifying the molecular predictors of rectal cancer's response to PCRT.^6,7^ In order to identify predictive biomarkers of responsiveness to PCRT, it is critical that we have a standardized classification of response. Tumor regression grade (TRG)—which scores the relative proportion of residual tumor to stromal fibrosis^8^—has been widely used to determine the level of tumor response to PCRT.

No consensus, however, has yet been reached regarding uniform standards for determining good indicators of response to PCRT. In many studies, patients with pathologic near-total regression (NTR) have been included in similar prognostic groups as patients with pathologic total regression (TR).^9,10^ While, several recent studies also report controversial results.^11,12^ The pilot analysis of our data showed significantly higher rates of recurrence in NTR patients than TR patients, which led us to question it is appropriate to consider NTR along with TR as an indicator of good response. This study evaluated the oncologic outcomes of patients with rectal cancer showing pathologic NTR on TRG after PCRT, and we compared these outcomes with TR patients in order to determine if NTR is an appropriate indicator of a good response to PCRT.

## METHODS

### Patients

We included 263 patients with locally advanced, primary, mid-, and low-rectal cancer located within 10 cm of the anal verge. All patients received PCRT followed by radical resection between January 2008 and December 2011 at Asan Medical Center, Seoul, Korea. Tumors were clinically diagnosed as T3/T4 or N+ on magnetic resonance imaging (MRI) and no evidence of distant metastasis on pretreatment work-ups was defined as locally advanced rectal cancer. Patients who were diagnosed with TR or NTR on pathologic examination of the resected specimens were included, while cases with other TRG, including no, minimal, or moderate regression was excluded in this study. Patients with a prior or concurrent malignancy were excluded. Prior to treatment, medical histories were thoroughly noted and physical examinations were used to assess all patients, including digital rectal examination, complete blood count, blood chemistry, measurement of carcinoembryonic antigen (CEA) concentrations, colonoscopy, chest radiography, computed tomography (CT) of the abdomen and pelvis, and pelvic MRI. Each patient provided informed consent before treatment.

### PCRT and Surgical Treatment

Preoperative radiotherapy consisted of 45 to 50 Gy administered in 25 fractions to the entire pelvis, followed by a 5.4-Gy boost to the primary tumor administered in 3 fractions. Chemotherapy was delivered as 2 cycles via an intravenous bolus of 5-fluorouracil (FU) (375 mg/m^2^ per d) and leucovorin (LV) (20 mg/m^2^ per d) over 3 days during the 1st and 5th weeks of radiation therapy, or oral capecitabine (1650 mg/m^2^ per d) was administered twice-daily during radiation therapy. For some patients, an oxaliplatin-based regimen or TS-1 was used. Radical surgical resection was planned for 5 to 8 weeks after completing PCRT. Surgical resection was performed according to the principle of total mesorectal excision.^13,14^

### Histopathologic Examination and TRG

Pathologic responses to PCRT were evaluated in the resected specimens using the TRG system suggested by the Gastrointestinal Pathology Study Group of the Korean Society of Pathologists.^15^ Tumor regression was scored using a 5-tier system: TR, total regression with no residual tumor cells and only fibrotic mass; NTR, near-total regression with microscopic residual tumor (ie, difficult to find) in the fibrotic tissue; moderate regression, dominant irradiation-related changes with residual tumor (ie, easy to find); minimal regression, dominant tumor mass with obvious irradiation-related changes; and no regression and no evidence of irradiation-related changes (fibrosis, necrosis, and vascular change). The grading system used in the present study can be easily translated to the Mandard and Dworak TRG system (see Table, Supplemental Content, which describes the 5-tier tumor regression grading system suggested by the Gastrointestinal Pathology Study Group of the Korean Society of Pathologists and compares it with other grading systems).

Pathologic stage (ypT and ypN) was determined according to the 7th AJCC TNM classification.^16^ Central review of the TRG was performed by 1 dedicated pathologist who specializes in colorectal malignancy. Patients with TR were classified into the TR group, and patients with NTR were classified into the NTR group.

### Follow-Up and Oncologic Outcomes

Postoperative follow-up consisted of physical examination, serum CEA measurement, and chest radiography every 3 to 6 months, plus abdominal pelvis and chest CT every 6 months to 1 year. Colonoscopy was performed at 6 months to 1 year postoperatively, and then every 2 to 3 years thereafter. Recurrence was determined according to radiological or histopathological findings. Local recurrence was defined as recurrence in the areas contiguous to the bed of the primary rectal resection or the site of anastomosis,^17^ and distant metastasis was defined as any recurrence outside of the pelvic cavity or dissemination to the peritoneal surface. Recurrence-free survival (RFS) was defined as the time between surgery and the first recurrence event or death.

### Statistical Analysis

The clinical characteristics were compared between the TR and NTR using Pearson Chi-squared test, Fisher exact test, or Student *t* test, as applicable. Survival curves were constructed using the Kaplan–Meier method and were compared using log-rank tests. The association between clinical factors and RFS were performed using Cox proportional hazard regression. To evaluate the summarized RFS rates of the published studies in the literature, which reported the RFS rates for the TR and NTR, a meta-analysis for summing up survival curve was performed using generalized mixed linear model.^18^ Here, *P* < 0.05 was considered significant for all analyses (SPSS ver. 21.0, IBM statistics, Armonk, NY).

## RESULTS

### Patient Characteristics and Pathologic Outcomes

The NTR group included more male patients; however, age, tumor location, sphincter preservation rate, and the administered combination chemotherapy regimens did not differ between groups. Neither pretreatment nor preoperative CEA demonstrated significant differences. Lymphovascular invasion and perineural invasion were only observed in the NTR group. Significantly more patients in the NTR group underwent adjuvant chemotherapy (Table 1). The NTR group demonstrated a heterogeneous ypT stage distribution, and most patients were ypT2 (n = 72, 55%). Lymph node metastases were more frequently present in the NTR group (24.4% in the NTR group vs 6.8% in the TR group; *P* < 0.001; Table 2).

**TABLE 1:**
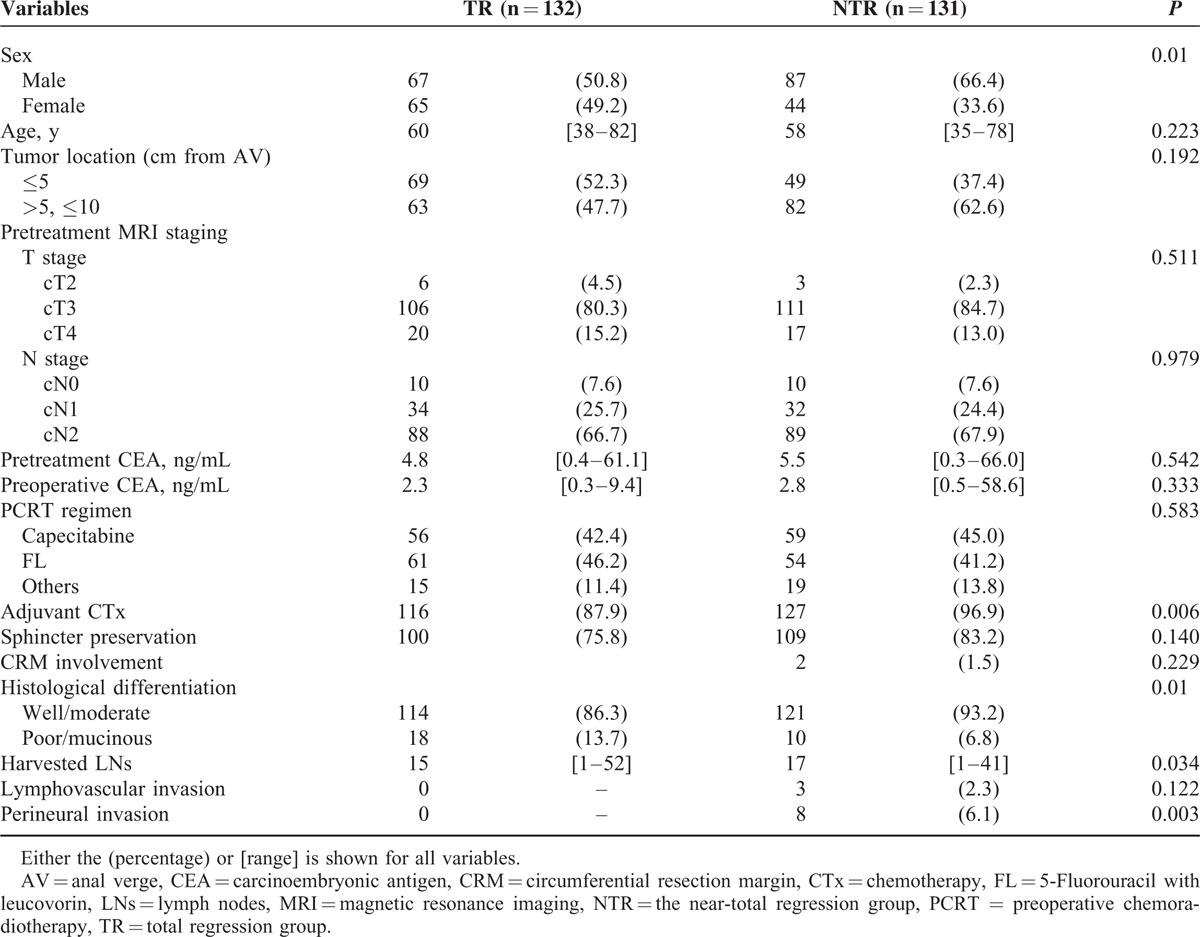
Clinicopathological Characteristics of the Study Patients

**TABLE 2:**
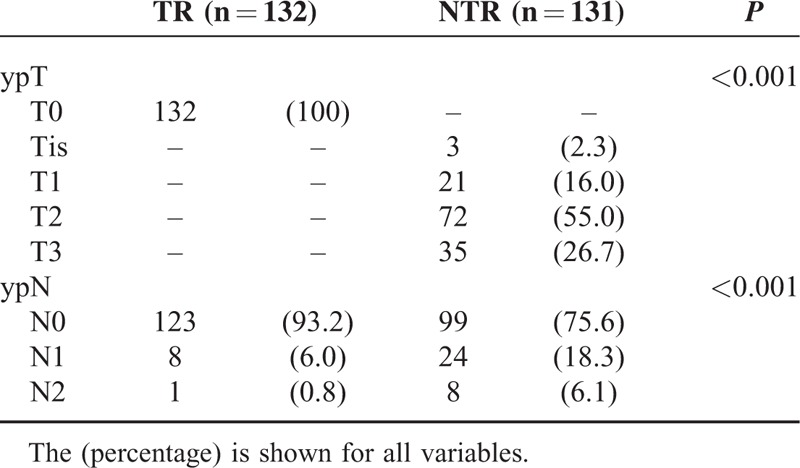
ypT and ypN Status in the Total Regression (TR) and the Near-Total Regression (NTR) Groups

### Oncologic Outcomes and Factors Associated With RFS

**FIGURE 1:**
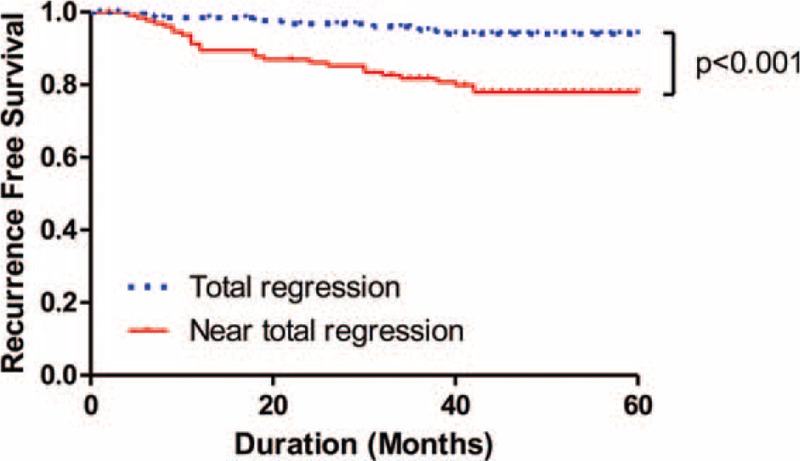
Five-year recurrence-free survival (RFS) rates of the total regression and the near-total regression groups. The total regression showed significantly higher 5-year RFS than the near-total regression.

**FIGURE 2:**
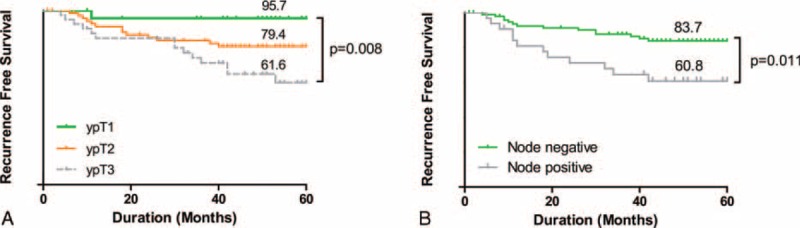
Five-year recurrence-free survival (RFS) rates according to ypT and ypN status patients in the near-total regression group. RFS was stratified according to ypT (A) and ypN (B) status.

The cumulative recurrence rate was 19.8% in the NTR group, which is significantly higher than 6.1% in the TR group (*P* = 0.003), over the mean follow-up periods of 41 and 42 months, respectively. A single local recurrence case was observed in the TR group, and 3 cases of local recurrence were identified in the NTR group (*P* = 0.53). There was a significant difference in the distant metastasis rate between groups: 8 cases in the TR group (6.1%) versus 25 cases in the NTR group (19.1%). The lung was the most frequent site of metastasis in both groups. The 5-year RFS rate was significantly higher in the TR group (94.0%) than the NTR group (77.8%; Fig. 1). TRG was confirmed as the only independent prognostic factor of RFS by the multivariate analysis (Table 3). Within the NTR group, the 5-year RFS rates were stratified according to ypT stage as follows: 95.7% for ypT1; 79.4% for ypT2; and 61.6% for ypT3 (Fig. 2A). In the NTR group, patients with metastatic lymph nodes (ypN+) demonstrated a significantly lower 5-year RFS rate than ypN-patients (Fig. 2B).

**TABLE 3:**
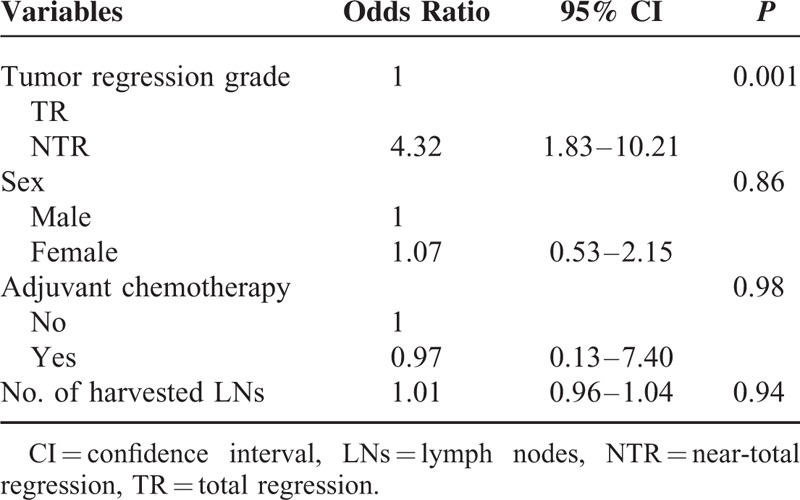
Multivariate Analysis of the Factors Associated With 5-Year Recurrence-Free Survival

### Summary of the RFS Data in the Published Literature

**FIGURE 3:**
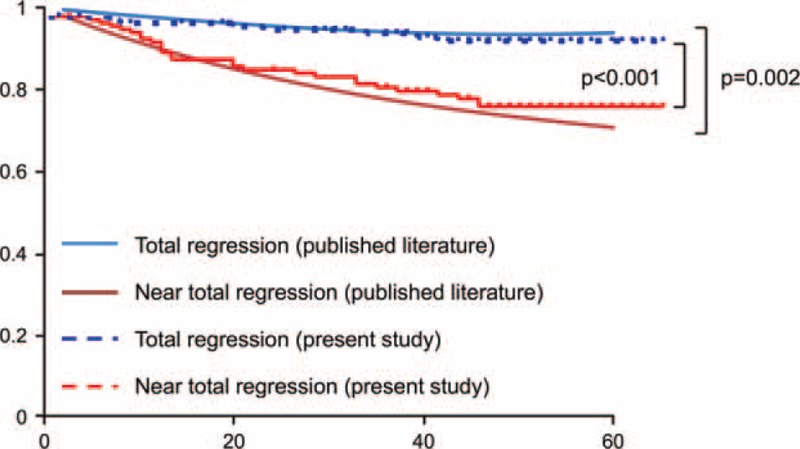
Comparison of the recurrence-free survival (RFS) in the present study with that of the published literature. Summary of the RFS rates in the published literature was assessed using generalized mixed linear model. Survival curves of each analysis showed similar difference between the total regression and the near-total regression groups.

We identified the RFS rates for TR and NTR in the published literature. Five studies were included in our analysis, each of which reports RFS rates for TR and NTR and included >20 enrolled cases.^3,11,12,19,20^ The summarized RFS curve of the 5 studies (determined using a meta-analysis with generalized linear mixed modeling) showed that NTR demonstrates significantly poorer RFS than TR (*P* = 0.002; Fig. 3). The differences in RFS between the TR and NTR groups in the summarized analysis are quite similar to the differences observed in the present study. The 5-year RFS of the TR and NTR were 94.2% versus 77.7%, and those in the present study were 94% versus 77.8%, respectively (Fig. 3).

## DISCUSSION

In the era of precision medicine, many research topics focus on discovery of predictive biomarkers that determine a patient's individual treatment strategy, regardless of therapeutic areas. It is not an exception in the field of colorectal cancer and it is a great unmet need that we have to find a way to predict individual PCRT response in patients with rectal cancer. Although many studies have been conducted to identify predictive markers of PCRT in rectal cancer,^21^ practical, applicable, and predictive markers have not been proposed. Lack of consensus on standardized classification of response might be 1 of the reasons because it is mandatory that we have a uniform standard to determine good or poor response that correlates with oncologic outcomes.

Until now, TRG had been widely used to determine the tumor response to PCRT.^3–5,19,22^ However, the classifications for a good responder to PCRT differs among researchers who use different TRGs^10,23–25^; some studies considered only TR as a good responder, while others included TR with NTR in the same response group. Therefore, on a practical level, consensus regarding good responders to PCRT must precede research. This discordance between studies may derive from the lack of clear pathological definitions for tumor response and inter-observer variability among pathologists. In the literature, different definitions of pathologic complete response have been reported.^11^ Strictly speaking, within the context of TRG, complete response only includes the status of the primary tumor, while others define a pathologic complete response as no residual disease at all (ie, ypT0N0). Furthermore, the lack of a clear pathological definitions for “fibrosis” leads to different interpretations using the TRG system, thereby leading to difficulties when assigning TRG.^26^ In our study, we questioned whether the current practice of regarding NTR as the same prognostic group as TR, as an indicator of good response to PCRT is appropriate or not. The results demonstrated a significantly higher recurrence rate and poorer RFS in the NTR group than the TR group, which suggests that NTR should not be equally considered as a good prognostic group as TR.

In many studies, patients with pathologic NTR are included in a similar prognostic group as patients with pathologic TR.^20,27^ In publications on the correlation between TRG and outcomes, NTR is often analyzed as a uniform group with TR. However, the number of patients is too small and different TRG systems are used in several studies; therefore, whether or not NTR should be included in the same prognostic group as TR remains inconclusive. In fact, a recent study assessed several TRG systems and reported a poor RFS rate in NTR group.^12^ Our present study also showed that the RFS of NTR overlaps with moderate regression—rather than TR—on both the Mandard and Dworak/Rodel systems. In our dataset, we found that the patients in the NTR group were classified with various ypT and ypN statuses. In NTR patients, the distribution of residual cancer cells within the bowel wall after PCRT and surgery is heterogeneous according to several studies.^4,28^ Metastatic lymph nodes were also more frequently found in the NTR group. The reason for the poorer outcomes in the NTR group may be the heterogeneous distribution, in terms of depth of invasion and lymph node metastasis, within this group. Actually, the RFS rates of the patients in the NTR group significantly differed depending on ypT and ypN status, as shown in Figure 2. A lower RFS rate among the patients with positive nodes—regardless of the TRG system—has also been reported, and the importance of pathologic node-negative status has been emphasized.^11^

In an effort to demonstrate a summarized data of the RFS rates of the TR and NTR from the published literature using meta-analysis, we identified 5 studies that report the RFS rates for TR and NTR, respectively.^3,11,12,19,20^ One of them was a meta-analysis of 14 studies.^3^ The RFS rates of each study were analyzed using generalized linear mixed modeling and we found that NTR had significantly poorer RFS than that of TR.^18^ Comparison of curves of our present study on the summarized RFS curves revealed similar differences between groups. Contrary to the impressions of each individual study, the RFS rate for NTR was significantly poorer than TR.

Due to the shortcomings inherent to any retrospective analysis, this study enrolled a heterogeneous patient population that received different preoperative and postoperative chemotherapy regimens, which could have interfered with the oncologic outcomes. The limitations to our study also included the lack of a long follow-up period. Considering the relatively high rate of late recurrence following PCRT, additional long-term follow-up studies are needed.

The use of different pathologic TRG systems is 1 of the causes of inconsistent results between studies. Here, we used the uniform TRG system suggested by the Gastrointestinal Pathology Study Group of the Korean Society of Pathologists, and this can be translated into other TRG systems such as Mandard and Dworak TRG. Therefore, the results of our present study have an advantage of getting a fair comparison with other studies. Inter-observer variability among pathologists is 1 of the most important factors that affect the results and quality of a study. In our present analyses, a highly trained, dedicated pathologist who specializes in gastrointestinal malignancy repeatedly graded the tumor responses by centrally reviewing the resected specimens in order to diminish shortcomings. This is relatively large-numbered study incorporating a central review that demonstrated prognostic differences between TR and NTR.

In conclusion, NTR demonstrates significantly poorer oncologic outcomes than TR. Therefore, consideration of NTR as an indicator of good response together with TR may not be appropriate. Future prospective studies with a larger number of patients and a longer follow-up duration should provide further subdivision and risk stratification among patients who are treated with PCRT in rectal cancer.

## Supplementary Material

Supplemental Digital Content

## References

[1] Benson AB III Bekaii-Saab T Chan E Rectal cancer. *J Natl Compr Canc Netw* 2012; 10:1528–1564. 23221790 10.6004/jnccn.2012.0158

[2] Habr-Gama A Perez RO Nadalin W Long-term results of preoperative chemoradiation for distal rectal cancer correlation between final stage and survival. *J Gastrointest Surg* 2005; 9:99–101.90–99; discussion. 10.1016/j.gassur.2004.10.010 15623449

[3] Maas M Nelemans PJ Valentini V Long-term outcome in patients with a pathological complete response after chemoradiation for rectal cancer: a pooled analysis of individual patient data. *Lancet Oncol* 2010; 11:835–844. 20692872 10.1016/S1470-2045(10)70172-8

[4] Swellengrebel HA Bosch SL Cats A Tumour regression grading after chemoradiotherapy for locally advanced rectal cancer: a near pathologic complete response does not translate into good clinical outcome. *Radiother Oncol* 2014; 112:44–51. 25018000 10.1016/j.radonc.2014.05.010

[5] Rodel C Martus P Papadoupolos T Prognostic significance of tumor regression after preoperative chemoradiotherapy for rectal cancer. *J Clin Oncol* 2005; 23:8688–8696. 16246976 10.1200/JCO.2005.02.1329

[6] Adlard JW Richman SD Seymour MT Prediction of the response of colorectal cancer to systemic therapy. *Lancet Oncol* 2002; 3:75–82. 11902527 10.1016/s1470-2045(02)00648-4

[7] Garcia-Aguilar J Chen Z Smith DD Identification of a biomarker profile associated with resistance to neoadjuvant chemoradiation therapy in rectal cancer. *Ann Surg* 2011; 254:486–492.483–492; discussion. 21865946 10.1097/SLA.0b013e31822b8cfa PMC3202983

[8] Dworak O Keilholz L Hoffmann A Pathological features of rectal cancer after preoperative radiochemotherapy. *Int J Colorectal Dis* 1997; 12:19–23. 9112145 10.1007/s003840050072

[9] Garcia-Aguilar J Hernandez de Anda E Sirivongs P A pathologic complete response to preoperative chemoradiation is associated with lower local recurrence and improved survival in rectal cancer patients treated by mesorectal excision. *Dis Colon Rectum* 2003; 46:298–304. 12626903 10.1007/s10350-004-6545-x

[10] Kim IJ Lim SB Kang HC Microarray gene expression profiling for predicting complete response to preoperative chemoradiotherapy in patients with advanced rectal cancer. *Dis Colon Rectum* 2007; 50:1342–1353. 17665260 10.1007/s10350-007-277-7

[11] Abdul-Jalil KI Sheehan KM Kehoe J The prognostic value of tumour regression grade following neoadjuvant chemoradiation therapy for rectal cancer. *Colorectal Dis* 2014; 16:O16–O25. 24119076 10.1111/codi.12439

[12] Trakarnsanga A Gönen M Shia J Comparison of tumor regression grade systems for locally advanced rectal cancer after multimodality treatment. *J Natl Cancer Inst* 2014; 106: dju248. 10.1093/jnci/dju248 PMC4271114 25249540

[13] Heald RJ Ryall RD Recurrence and survival after total mesorectal excision for rectal cancer. *Lancet* 1986; 1:1479–1482. 2425199 10.1016/s0140-6736(86)91510-2

[14] Kapiteijn E van De Velde CJ European trials with total mesorectal excision. *Semin Surg Oncol* 2000; 19:350–357. 11241917 10.1002/ssu.5

[15] Chang HJ Park CK Kim WH A standardized pathology report for colorectal cancer. *Korean J Pathol* 2006; 40:193–203.

[16] Edge S Byrd D Compton C AJCC Cancer Staging Manual. 7th ed New York: Springer; 2010.

[17] Gordon P Nivatvongs S Principles and Practice of Surgery for the Colon, Rectum, and Anus. 2nd ed St Louis, MO: Quality Medical Publishing, Inc.; 1999.

[18] Arends LR Hunink MGM Stijnen T Meta-analysis of summary survival curve data. *Stat Med* 2008; 27:4381–4396. 18465839 10.1002/sim.3311

[19] Bujko K Kolodziejczyk M Nasierowska-Guttmejer A Tumour regression grading in patients with residual rectal cancer after preoperative chemoradiation. *Radiother Oncol* 2010; 95:298–302. 20430458 10.1016/j.radonc.2010.04.005

[20] Lim SB Yu CS Hong YS Long-term outcomes in patients with locally advanced rectal cancer treated with preoperative chemoradiation followed by curative surgical resection. *J Surg Oncol* 2012; 106:659–666. 22674581 10.1002/jso.23181

[21] Akiyoshi T Kobunai T Watanabe T Predicting the response to preoperative radiation or chemoradiation by a microarray analysis of the gene expression profiles in rectal cancer. *Surg Today* 2012; 42:713–719. 22706722 10.1007/s00595-012-0223-8

[22] Hermanek P Merkel S Hohenberger W Prognosis of rectal carcinoma after multimodal treatment: ypTNM classification and tumor regression grading are essential. *Anticancer Res* 2013; 33:559–566. 23393349

[23] Ghadimi BM Grade M Difilippantonio MJ Effectiveness of gene expression profiling for response prediction of rectal adenocarcinomas to preoperative chemoradiotherapy. *J Clin Oncol* 2005; 23:1826–1838. 15774776 10.1200/JCO.2005.00.406 PMC4721601

[24] Pucciarelli S Rampazzo E Briarava M Telomere-specific reverse transcriptase (hTERT) and cell-free RNA in plasma as predictors of pathologic tumor response in rectal cancer patients receiving neoadjuvant chemoradiotherapy. *Ann Surg Oncol* 2012; 19:3089–3096. 22395986 10.1245/s10434-012-2272-z

[25] Huh JW Lee JH Kim HR Pretreatment expression of 13 molecular markers as a predictor of tumor responses after neoadjuvant chemoradiation in rectal cancer. *Ann Surg* 2014; 259:508–515. 23787217 10.1097/SLA.0b013e31829b3916

[26] Bateman AC Jaynes E Bateman AR Rectal cancer staging post neoadjuvant therapy—how should the changes be assessed? *Histopathology* 2009; 54:713–721. 19438746 10.1111/j.1365-2559.2009.03292.x

[27] Kuo LJ Liu MC Jian JJ Is final TNM staging a predictor for survival in locally advanced rectal cancer after preoperative chemoradiation therapy? *Ann Surg Oncol* 2007; 14:2766–2772. 17551794 10.1245/s10434-007-9471-z

[28] Duldulao MP Lee W Streja L Distribution of residual cancer cells in the bowel wall after neoadjuvant chemoradiation in patients with rectal cancer. *Dis Colon Rectum* 2013; 56:142–149. 23303141 10.1097/DCR.0b013e31827541e2 PMC4674069

